# Steepening of inertial Alfvén waves

**DOI:** 10.1017/S0022377826101330

**Published:** 2026-03-05

**Authors:** Ian DesJardin, John Dorelli, Lynn Wilson, George Khazanov, Jason Shuster

**Affiliations:** 1 NASA Goddard Spaceflight Center, Greenbelt, MD 20771, USA; 2 Department of Physics, The Catholic University of America, 620 Michigan Ave NE, Washington, DC 20064, USA; 3 Institute for the Study of Earth, Oceans, and Space, University of New Hampshire, Durham, NH 03824, USA

**Keywords:** space plasma physics, plasma nonlinear phenomena, plasma waves

## Abstract

Inertial Alfvén waves are thought to accelerate electrons to auroral energies via their parallel electric field in the Earth’s magnetosphere. During active geomagnetic times, it is estimated that a significant percentage of electron precipitation energy into the Earth’s ionosphere can be attributed to these waves. However, self-consistent wave/particle interactions of inertial Alfvén waves with the accelerated electron population are not well understood. We show that recent self-consistent models have a strong nonlinearity in them. A reduced set of equations which describe this nonlinear steepening is derived and shown to agree with drift-kinetic simulations and other published studies. From this reduced set of equations, many properties of the nonlinearity are derived and shown to agree with simulations. This includes the time and length scales and connecting the speed of the wave to the perturbation maximum value.

## 1. Introduction

Acceleration of electrons to keV energy by inertial Alfvén wave activity can account for substantial portions of ionospheric energy deposition at local midnight, especially during storm times (Chaston *et al*. 2007, 2008). This type of activity is inferred by the presence of time-dispersion electrons on the edges of or sometimes coincident with inverted-V distributions (Feltman *et al*. 2025). Inertial Alfvén waves differ from magnetohydrodynamic (MHD) Alfvén waves when the perpendicular wavelength goes below the electron skin depth ( 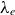 ). Under these conditions, the electron physics decouples from the ions at MHD scales and a parallel electric field ( 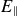 ) can be supported in the plasma. The spatial structure of the aurora is also believed to be determined by this physics. Transient optical features are often observed with thicknesses near 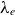 which matches the spatial structure of inertial Alfvén waves (Stasiewicz *et al*. 2000).

Properties of electron precipitation driven by the parallel electric field of the wave can depend on the field-aligned wave shape. A sinusoidal wave will have a parallel electric field that is equal parts positive and negative, while a steepened wave will have an asymmetric electric field near the sharpest gradient. Kletzing (1994) showed that the acceleration of test particles in an Alfvén wave could be reproduced by one-bounce Fermi acceleration. Under this model, the shape of the wave does not effect the velocities of the accelerated particles nor their final energy. However, it does change the region of the wave where the acceleration happens resulting in a spectrally narrower suprathermal population in an energy–time spectrogram. The final energy of non-one-bounce Fermi acceleration mechanisms will depend on the sharpness of the wave.

Steepening of the inertial Alfvén waves has been observed several times in the literature (Hui & Seyler 1992; Rankin & Tikhonchuk 1998; Watt & Rankin 2007, 2008, 2010; Seyler & Liu 2007). Hui & Seyler (1992) and Seyler & Liu (2007) identify in text steepening as a phenomenon pertinent to electron acceleration and transverse acceleration of ion events. Other studies demonstrate steepening without directly pointing it out. Andersson *et al*. (2002) shows Freja data that appear to have a much sharper gradient on the leading edge than the trailing edge of the irregularity. Damiano *et al*. (2005) appears to have steepening present in the simulation results. Swift (2007*a*) shows a much smaller length scale of the parallel electric field than the potential on only one side of it. Especially direct, Goertz & Boswell (1979) note that parallel electric fields, ‘exist only on the leading edge of an oblique electrostatic shock’.

Several previous works explicitly focus on nonlinear solutions (Wu & Chao 2004; Dubinin, Sauer & McKenzie 2005). Our solutions somewhat resemble the inertial Alfvén soliton in figure 7 of Dubinin *et al*. (2005). However, these cited works do not capture the time and spatial scales of wave steepening.

It is interesting that given the importance of Alfvénic acceleration, not much detail has been enunciated to describe the steepening of inertial Alfvén waves. In this study, we apply the method of characteristics to solve for jump conditions, length and time scales of wave steepening and compare them with the numerical solution from drift kinetics. We find good agreement between the method-of-characteristic-derived steepening and our simulation results. These techniques allow for analytical understanding of the nonlinear time and length scales.

## 2. Methodology

This study follows the work of Watt, Rankin & Marchand (2004) to simulate a gyrotropic electron distribution function ( 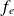 ). The distribution function is directly simulated in independent coordinates 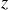 (distance along the background magnetic field line), 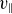 and 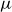 (magnetic moment). The basic assumptions that go into such a model are that the perturbations have a large aspect ratio ( 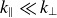 ) such that the magnetic perturbations can be captured with a single scalar potential ( 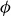 ) and only the parallel component of the vector potential ( 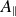 ), the parallel current is given by the electrons and the perpendicular current is given by the ion polarisation drift (Kadomtsev 1965; Streltsov & Lotko 1995). The electrons are evolved by the drift-kinetic equation
(2.1)

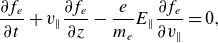




where 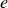 is the elementary charge and 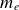 is the electron mass. Magnetic moment 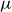 is conserved in this model. The parallel electric field is given by
(2.2)

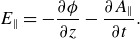

It is assumed that there is no perpendicular component of the vector potential because there is no compression of the magnetic field 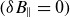 . All quantities vary 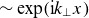 in the perpendicular direction, where 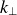 is constant. With these assumptions, Ampère’s law takes the form of
(2.3)

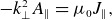



(2.4)

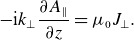




The perpendicular current is given by the ion polarisation current 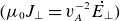 . Substituting this into (2.4), one finds that the field evolves like
(2.5)

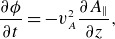




where 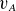 is the Alfvén speed. Equations (2.1), (2.2) and (2.5) form a self-consistent set. Here 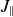 is solved by taking the first moment of the electron distribution function at every time step using trapezoidal quadrature. Equation (2.5) is only valid for a wave of constant perpendicular wavenumber independent of altitude (Mottez 2013; Watt & Rankin 2013). This constrains our results to short distances along the magnetic field line and time scales shorter than perpendicular wave–wave interaction times. A more complete treatment would require one to resolve the perpendicular direction in the simulation domain (Swift 2007*a*, *b*).

The density and temperature of the background plasma are set to be constant. The goal of this study is to understand the nonlinearity in isolation from these other effects. Certainly in the real magnetosphere, these variations will change the result. However, the time scales for this nonlinearity are less than a second. This nonlinearity may happen faster than the wave can propagate, which justifies this analysis. The background plasma is a hydrogen plasma with conditions 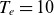 eV, 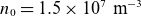 and 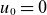 . The background magnetic field is straight and uniform with amplitude 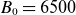 nT. This makes the Alfvén speed along the domain a uniform 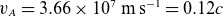 . The perpendicular scale is 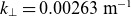 . The electron inertial length 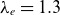 km and 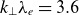 . The domain is of length 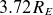 with periodic boundary conditions, and the simulation is run to 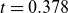 s. The disturbances never reach the boundary during this time period. The initial conditions is a Gaussian in 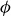 with amplitude −100 V and width 0.07 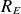 ( 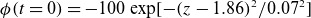 ). The other quantities are set to the background conditions. These parameters represent the Earth’s auroral zone at an altitude of 7000 km ( 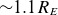 ) (Kletzing 1994).

A pseudo-spectral numerical method is used for the simulation. In 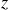 an equal spaced Fourier basis is used with 256 points. Simulations with 512 and 1024 points were also conducted, but they give the same basic result. In 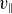 and 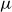 a Chebyshev basis is used with, respectively, 128 and 64 points. Differentiation of 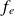 is done by matrix–tensor products using the pseudo-spectral differentiation matrix for each basis (Fornberg 1996; Trefethen 2000). Our code is implemented in Python. The cupy library (Okuta *et al*. 2017) is used to accelerate the computation on a graphical processing unit. The distribution function is integrated in time with a fourth-order Runge–Kutta method. Potential 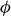 is integrated after each Runge–Kutta time step with a forward Euler integration. The drift-kinetic equation has an explicit dependence on 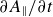 . Other works move to different variables in order to avoid this complication (Watt *et al*. 2004). This study uses Picard iteration to resolve each time step and refine the instantaneous 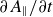 . Three Picard iterations are used per time step. A variable time step is used depending on the advection coefficients of (2.1) calculated at each time step.

## 3. Results

### 3.1. Drift-kinetic simulation

**Figure 1.**
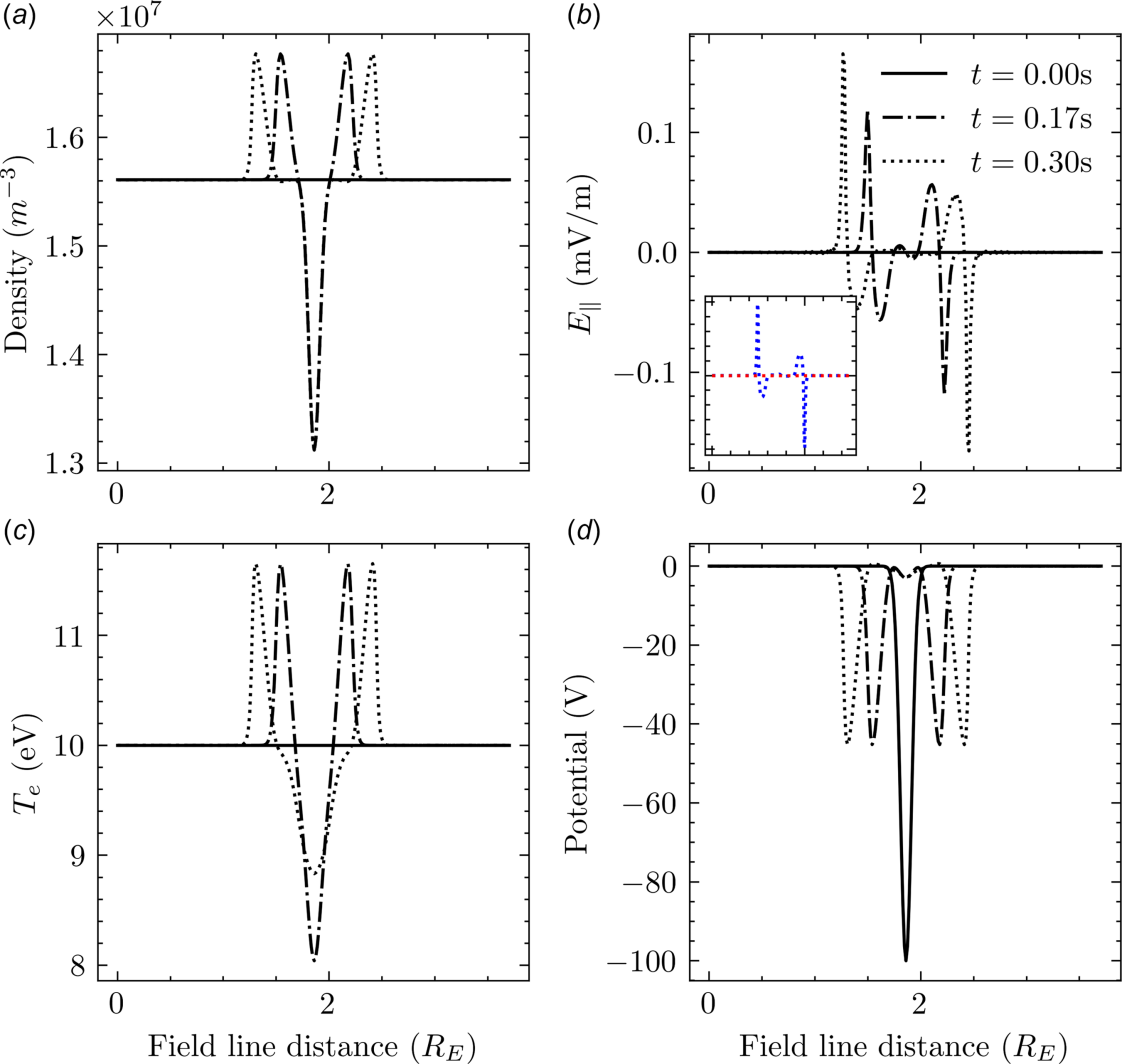
(*a*–*d*) Drift-kinetic simulation results. The legend for all subplots is in (*b*). The inset in (*b*) shows the constituent parts of 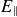 at 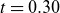 s. The red line indicates 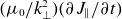 and the blue line indicates 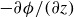 . The *x* and *y* axes of the inset are identical to those of the other plots.

Using the simulation method described in § 2, figure 1 shows the output along a field line. Fluid moments are determined by integrating over the electron distribution function. Very quickly, two propagating disturbances move left and right in the simulation. These disturbances have variations in density, temperature and electrostatic potential. At 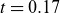 s the initial disturbance is present in figure 1. These initial conditions and results are similar to those used in Watt *et al*. (2004) (particularly compare our figure 1 with their figures 3 and 4).

While not shown in figure 1, the parallel current and electron velocity have similar shapes to the density and temperature. They are not shown directly but are used in later calculations.

As the disturbance propagates it begins to nonlinearly steepen. This can be seen in the shape of figure 1(*a*,*c*,*d*). The parallel electric field is determined in part by the gradient of the electrostatic potential (2.2). From figure 1 it can be seen that 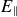 is predominantly electrostatic. It has a maximum out of phase with the main disturbance (see figure 1 *b*). As the disturbance nonlinearly steepens, the parallel electric field steepens in the parallel direction. The leading edge of the wave in the direction of propagation becomes substantially larger than the trailing edge. This can be seen in the change in shape of figure 1(*b*) from 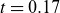 to 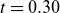 s. The electron temperature also follows this trend. Joule heating of the plasma caught in the wave happens due to the localised parallel electric field on the leading edge.

Unlike Watt *et al*. (2004), we do not directly observe an accelerated electron population. This is because the simulation is not run long enough to accelerate electrons. The reason for this is numerical. After 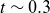 s, Gibbs phenomena appear around the discontinuity in both the field quantities and 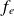 . The numerical solution quickly blows up and becomes unphysical. The Fourier transform of the parallel electric field reveals that the Gibbs phenomena happen when the wave steepens to the grid scale. In order to successfully accelerate electrons in the simulation, there must be a change to the numerical methods in order to stably integrate in time (2.1) in the presence of a discontinuity. It is expected that below grid scale, there is a dissipation mechanism or the wave collapses invalidating the one-dimensional approach. In either case, steepening still occurs and the results from before the subgrid gradient occurrence can be used to validate a model of the steepening.

**Figure 2.**
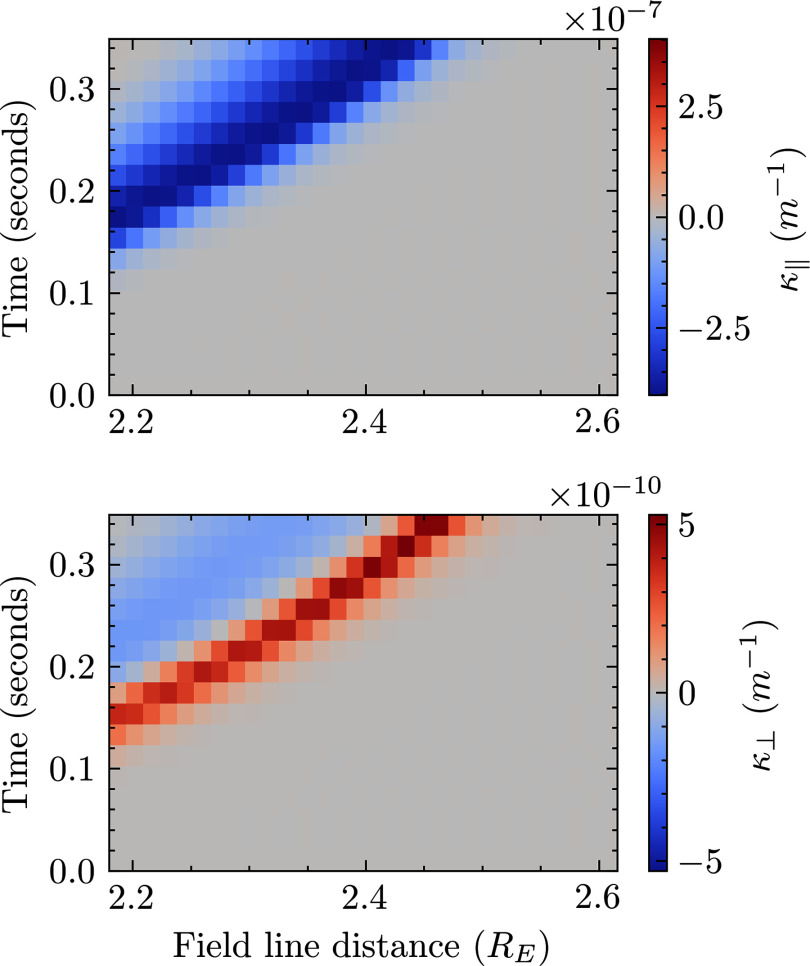
Curvature of field line from simulation results. The top panel indicates the curvature parallel to the background field line. The bottom panel indicates the curvature perpendicular to the background field line.

As is noted later, a key aspect of the physics is that curvature of the field line is increasing. Along the field line, the curvature can be measured as
(3.1)

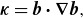




where 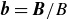 . Figure 2 shows the result of (3.1) calculated using the simulation results. Curvature 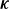 is shown in vector form with a parallel and perpendicular component. The result is zoomed in to the right-going disturbance. It can be seen that as time goes on in the simulation, the maximum curvature of the field increases. This indicates steepening. For 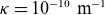 , the smallest radius of curvature is 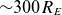 . Likewise, for 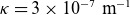 , the radius of curvature is 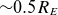 .

### 3.2. Derivation

We seek to formulate a simple model for the nonlinear propagation of inertial Alfvén waves. For inertial Alfvén waves, the pressure term is discarded from the electron momentum equation (Stasiewicz *et al*. 2000). Taking the first moment of (2.1) under low- 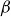 assumptions, the parallel current can be written as (Thompson & Lysak 1996)
(3.2)

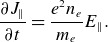




This neglects ion effects and corrections from finite electron pressure. Equations (3.3) and (3.4) are the set of coupled equations that result from combining (2.2), (2.3), (2.5) and (3.2):
(3.3)

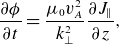



(3.4)

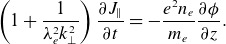




Taking the time derivative of (3.3) and the spatial derivative of (3.4), combining the mixed derivative term, and selecting the first-order terms it is found that
(3.5)

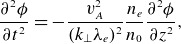




where 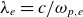 is the electron skin depth, 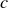 is the speed of light and 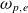 is the electron plasma frequency. The neglected terms are small under the conditions 
(3.6)

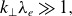



(3.7)

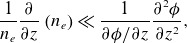



(3.8)

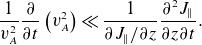




The first condition is true because of the focus on inertial Alfvén waves. The density and potential are proportional to each other in the results. Given this fact, the second condition (3.7) is simply a statement that the length scale of the first derivative is longer than the length scale of the second derivative. From figure 1, this can be seen to be true. The last condition (3.8) is true when the parallel wavelength is shorter than the variation in the background Alfvén conditions. Under the idealisation of straight homogeneous field lines, as in our simulation, this is true. Later in this study, the variation of the Alfvén speed along the field line is compared with the steepening length scale. When the steepening happens faster than the field line changes, the third condition is justified.

A relation between the perturbed electron density and the electrostatic potential is needed. For dispersive Alfvén waves, Hollweg (1999) (see (34)) has shown that the linearised density response is given by
(3.9)

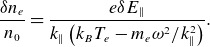




Under the conditions that 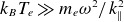 and that 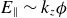 , (3.9) reduces to a Boltzmann response for the electron density, as is assumed in previous nonlinear treatments of kinetic Alfvén waves (e.g. Hasegawa & Mima 1976). However, for the case considered in this study, the first assumption is violated. The second term in the denominator of (3.9) will dominate, and hence the density response will depend on the frequency of the wave. In order to find a quasi-linear solution to this, the linearised dispersion relation
(3.10)

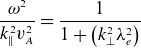




is used. Combining this with an assumption that the electrostatic part of the parallel electric field dominates, a simplified density response is given by
(3.11)

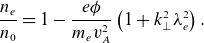

For the simulation results below, the maximum density increase is 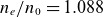 . The potential is 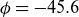 V and 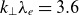 . Equation (3.11) predicts 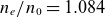 which is 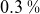 different from the simulation results. A Boltzmann relation predicts 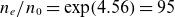 . This shows that (3.11) appropriately captures the density response of the electrons and that a Boltzmann response is a poor approximation. For a kinetic Alfvén wave ( 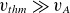 ) a Boltzmann response would be appropriate.

Next (3.11) is substituted into (3.5) while neglecting the electromagnetic term to form a simple model of the nonlinearity. Let 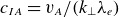 be the propagation speed. This is consistent with the well-known linear dispersion relation (Stasiewicz *et al*. 2000). This is lower than 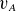 depending on the perpendicular wavelength. Then (3.5) can be written as
(3.12)

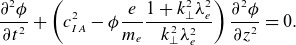




This wave equation exhibits a classical nonlinearity where the wave speed is modified by the wave amplitude. The modification is approximately 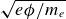 which is the maximum speed of a particle trapped in the potential of the wave. The term 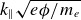 is the bounce frequency of a particle caught in the wave. The results of this modification to the linear phase speed are that nonlinear steepening occurs. Eventually a shock in 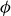 and 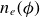 forms.

The wave speed in (3.12) only depends on 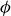 , so it can be decomposed into right- and left-going first-order wave equations via d’Alembert’s formula (Whitham 1974). The right-going wave equation is
(3.13)

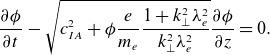




In the case that the wave is weakly nonlinear, which is defined here as 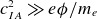 , and 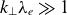 , then this can be further approximated as
(3.14)

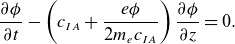




This derivation is meant to describe small-amplitude waves which are nonlinearly steepened until they have a large amplitude. Since the waves start small, using this weakly nonlinear assumption is justified in understanding the growth process. Equation (3.14) can be written in a weak form as
(3.15)

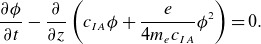

The term in parentheses is the flux of the wave. Equation (3.15) describes how the wave flux of this system depends on the local amplitude.

## 4. Analysis

### 4.1. Method of characteristics on derived partial differential equation

The weak form (3.15) and in particular knowledge of the flux allow the construction of a conservation law over the shock. In the frame of the shock moving at 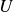 , where 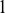 and 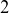 denote the upstream and downstream conditions, a shock will move at a speed given by the difference of the wave fluxes over the difference in wave amplitude ((2.18) in Whitham (1974)):
(4.1)

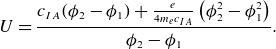

The Mach number is specific to a perpendicular wavenumber and is not the same as the Alfvénic Mach number. This construction is strictly only valid when the upstream and downstream conditions are constant around the discontinuity. In the simulation results, the downstream conditions are constant, but the upstream conditions are not. We assume that if the downstream conditions are 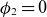 , then 
(4.2)

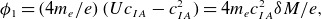



(4.3)

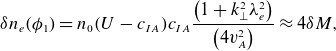




where 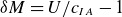 . In the magnetospheric plasma, it is a reasonable condition that in the ambient 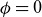 . In this test case 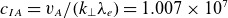 m s^−1^. For 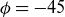 V, (4.2) predicts that the shock should have a propagation speed of 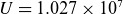 m s^−1^ and 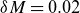 .

Using the locations of the wave at 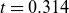 and 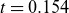 s, the wave speed is estimated from the simulation to be 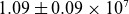 and 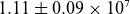 m s^−1^ tracking the maximum amplitude and midpoint of the discontinuity, respectively. If 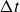 and 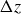 denote the time difference between snapshots ( 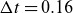 s) and grid size, respectively ( 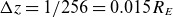 ), then the error in velocity could be estimated as 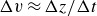 . This gives 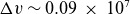 m s^−1^. To within the precision of this measurement, this agrees with the predicted propagation speed corresponding to the observed density and electrostatic potential via (4.2) and (4.3).

Equation (3.15) captures the minimal physics of the nonlinearity. Using the method of characteristics additional analytical properties of the solutions can be constructed. Consider an initial condition 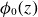 . Let 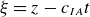 be the moving coordinate at the linear wave speed. The equations for the characteristics in time space diagrams are
(4.4)

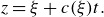




The shock forms when two characteristics overlap. Up until the solution becomes multi-valued, 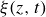 is defined implicitly from (4.4). Consider a Gaussian initial condition such as 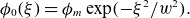 The disturbance fully steepens when the maximum speed characteristic 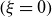 reaches the minimum characteristic ( 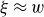 ). From (3.14) the maximum speed characteristic goes 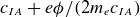 while the minimum speed characteristic goes 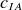 . Equating their characteristics (equation (4.4)), we find that
(4.5)

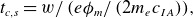



(4.6)

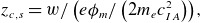




where 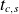 and 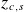 are the time and distance from the initial position where the characteristics intersect and hence the shock forms. They are the characteristic time and length scales over which nonlinearity becomes important. For 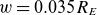 and 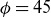 V as in our simulation, (4.5) and (4.6) respectively predict that 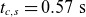 and 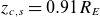 . These values are consistent with the time and length scales observed in the simulation. Both equations contain a factor of 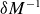 (defined in (4.3)). Of course, this simply denotes that a faster shock will steepen quicker. The amplitude of the wave ( 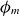 ) sets the time scale of the steepening as well as the propagation speed.

A more sophisticated estimate of the characteristic time and spatial scales of nonlinearity can be made by asking for the characteristic defined by the initial value 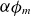 where 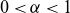 . This characteristic starts at 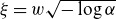 . The intersection time and space locations for a Gaussian are
(4.7)

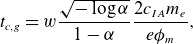



(4.8)

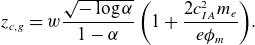




For the same set of parameters and setting 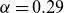 , 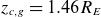 and 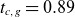 s. One can see that without the dependence on 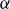 , (4.7) closely resembles (4.5) and (4.8) resembles (4.6). The function 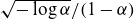 is convex. Near the maximum of a Gaussian, the characteristics have very similar slopes so it takes longer for them to collide. Far from the peak, the slopes are most different, but there is a longer distance separating the characteristics. The minimum of 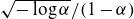 corresponds to the first characteristic that will intersect with the maximum speed characteristics by balancing a difference in slope with nearness to the peak. The minimum of 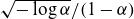 is 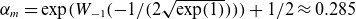 , where 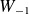 is the Lambert *W* function. This justifies our use of 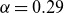 earlier. Numerically, 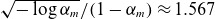 . This is the factor by which 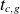 varies from 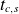 . Symbolic computations of this minimum can be found in the supplementary material.

Figure 1 shows that the wave appears very strongly steepened with an asymmetric shape and sharp leading edge at 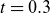 . In principle, one could compare the time of the shape change of the wave with (4.5) and (4.7). From inspection, it is clear that they are of the same order of magnitude. Perhaps there is a more precise way to do this comparison, but it is not explored in this study. Our analytical expressions for the characteristic nonlinear steepening time should be treated as very approximate. The factor of 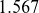 between (4.7) and 4.5 depends on the shape of the initial distribution. One would expect that other ‘shape factors’ may exist. Additionally, the initial left- and right-going waves are overlapping in the initial condition. This prevents this study from exactly articulating what the initial width and amplitude are.

**Figure 3.**
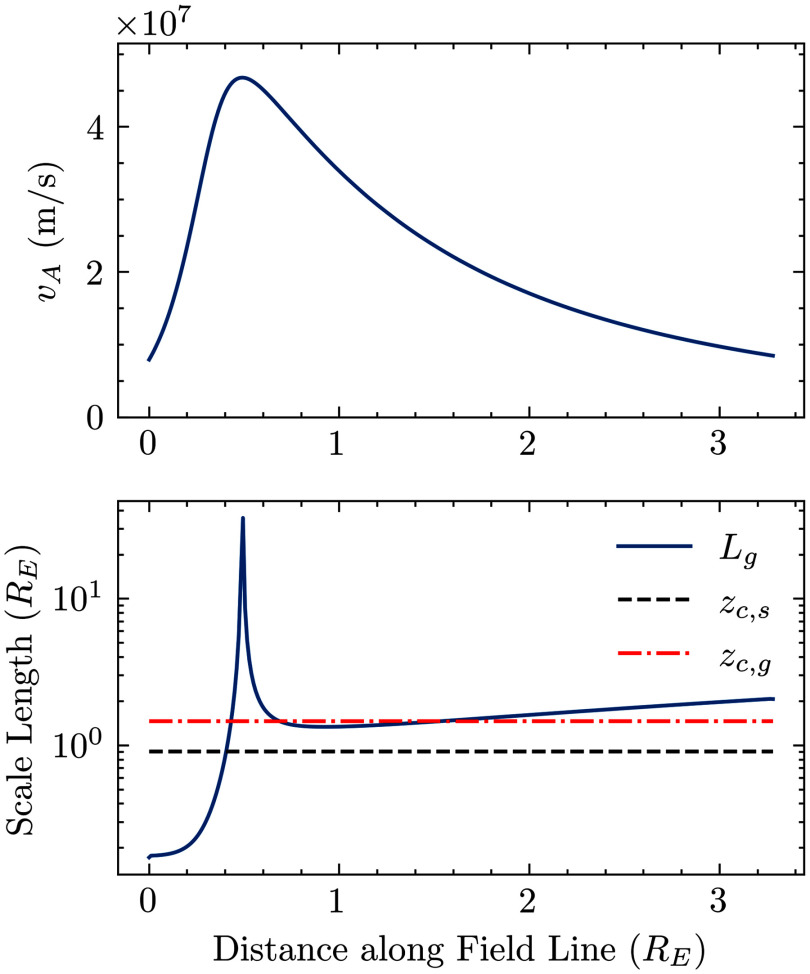
Comparison of the parallel gradient scales of the Alfvén speed with the length scale over which the wave steepens. The parallel Alfvén speed gradient ( 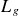 ) is defined in (4.9). The steepening length scales are defined in (4.6) and (4.8).

If the spatial scale of the nonlinearity is smaller than the spatial scale over which the Alfvén speed changes, it is justified to use a constant magnetic field and density to explore the physics of the nonlinearity in isolation. The strongest claim that can be made from those results is that the steepening will happen quickly enough to manifest in the real space plasma. Of course, it would be premature to then use those results to claim the actual shape of the wave. The time and spatial scales of the nonlinearity are estimated by (4.6) and (4.8). Figure 3 shows the Alfvén speed for a field line along *L* = 8.5 from a minimum altitude of 1000 km using a semi-empirical model of the plasma density at high latitudes. The quantity
(4.9)

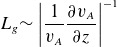

is a representative length scale for the Alfvén speed gradient. The two effects are often of the same order of magnitude. Above 0.4 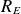 , the nonlinearity largely happens over a smaller distance than the Alfvén speed changes. Below 0.4 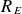 , the Alfvén speed changes on a shorter distance than the nonlinearity.

The constant density ( 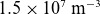 ), magnetic field (6.5 μT) and Alfvén speed ( 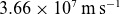 ) in this study correspond to 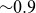 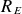 , where 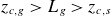 . The nonlinearity happens at a slightly smaller scale than the Alfvén speed gradients. This justifies the use of constant plasma parameters to simulate the steepening.

### 4.2. The MHD Rankine–Hugoniot conditions

For a slow-mode MHD shock where the normal direction of the shock is along the upstream magnetic field line, one of the jump conditions is (Kantrowitz & Petschek 1964)
(4.10)

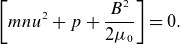




**Figure 4.**
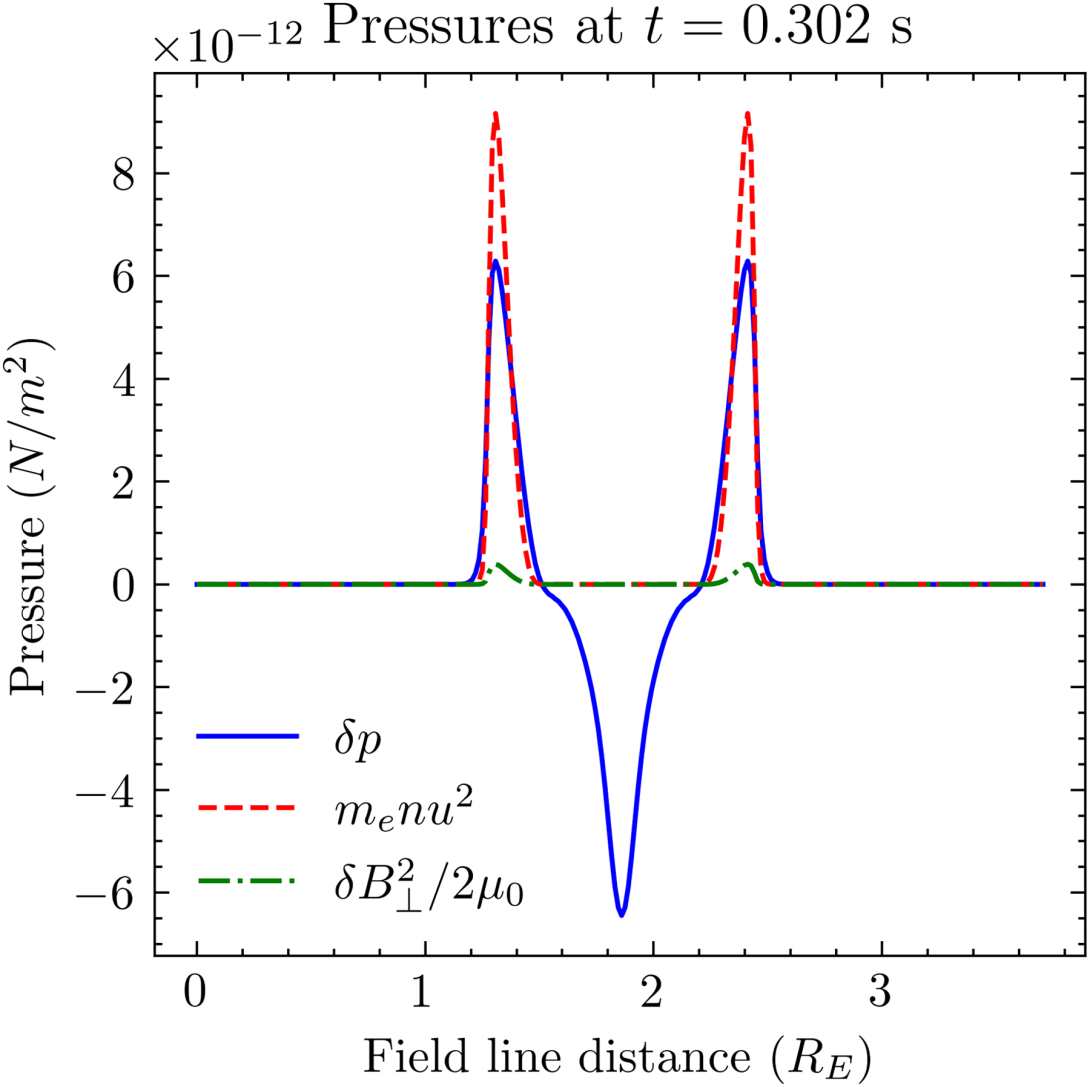
Pressure balance across the shock structure. The blue, green and yellow lines indicate, respectively, the difference in static pressure, dynamic pressure and magnetic pressure.

This is simply a statement of conservation of momentum flux density. The brackets indicate subtracting the upstream from the downstream conditions. The values for these terms in the jump condition are plotted in figure 4. In the frame of the shock, the downstream pressure is given by the sum of the dynamic pressure 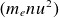 and free-stream static pressure 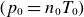 . The upstream pressure is given by the increase in static pressure 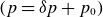 and the magnetic pressure 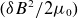 . In the inertial frame, as plotted, the increase in dynamic pressure is carried with the wave. As seen in figure 4, the maximum dynamic, difference in static and magnetic pressures are approximately 9.1, 6.3 and 0.4 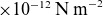 . The free-stream static pressure 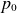 is 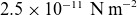 . The free-stream magnetic pressure, 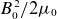 , is 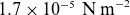 . These background pressures are, respectively, one and six orders of magnitude larger than any of the perturbations. The ratio of the free-stream static and magnetic pressures gives the plasma beta to be small, as is expected for an inertial Alfvén wave. It is interesting that the magnetic pressure appears to play a substantially smaller role in conservation of momentum than the dynamic pressure. The shock is almost hydrodynamic.

To within 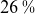 , figure 4 shows that the numerical solution satisfies (4.10) around the shock structure. At 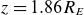 , there is a density depletion which is not balanced by the jump condition. Since this region is not across the discontinuity, other physics apply there. The pressures are calculated from fluid quantities which are themselves calculated from integrals of the distribution function using a trapezoid method. The velocity grid space is 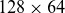 in parallel velocity, magnetic moment coordinates. There are errors present from the integration method which may explain the 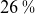 error. The free-stream plasma density as calculated by this method is 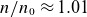 , instead of 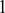 . Assuming a numerical error of 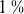 in each quantity, it is possible to see how a 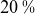 increase in density and temperature could be inaccurate by 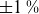 . Multiplied by other fluid quantities that are also inaccurate, a 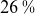 error in the jump conditions is possible. This is particularly so when 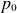 is one order of magnitude greater than the static pressure variation. One would expect this to give a 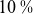 error in static pressure from a 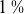 integration error. A higher-order integration method or a higher-resolution simulation would reduce this error.

The tangential component of the magnetic field 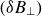 is non-zero downstream of the shock, but is zero upstream. Given this circumstance, it would be appropriate to draw a parallel with a switch-off slow-mode shock. In this instance, 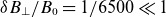 . The angle of the switch-off is given by 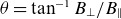 , which for this shock is very small. For a low- 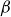 nearly parallel switch-off shock, it can be treated as an ordinary hydrodynamic shock (Kantrowitz & Petschek 1964). A normal hydrodynamic shock is described by
(4.11)

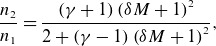



(4.12)

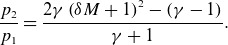




In the simulation results 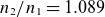 and 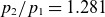 . For a value of 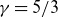 and 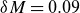 , (4.11) and (4.12) predict values of 1.08 and 1.24, respectively. This value of the Mach number is near the value of 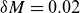 calculated via the jump conditions derived by the simplified nonlinear partial differential equation earlier (3.15) and (4.3).

The sound speed of this shock wave is not the magnetosonic speed. Rather, it is given by the linear phase speed of the inertial Alfvén wave!

### 4.3. Dissipation scales

#### 4.3.1. Breakdown of drift kinetics

A shock also forms in 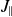 . When the perpendicular current is given by the ion polarisation drift, this shock predicts that the perpendicular electric field will also infinitely change in time. There must be a scale at which the assumptions of the perpendicular dynamics being entirely driven by the polarisation current must break down.

Equation (3.15) resembles the Burgers equation without any dissipation. We hypothesise that previous numerical work has implicitly had artificial dissipation present in the numerical scheme. This prevents the solution from infinitely steepening. However, it has little to do with the physical source of dissipation.

We hypothesise that the breakdown of drift kinetics could be modelled as a dissipation mechanism that triggers when the solution becomes steepened. Table 1 lists the order of magnitude of the pertinent length scales in the problem. Width 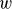 is the parallel width of the Alfvén wave, 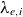 are the skin depths, 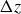 is the grid scale and 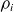 is the ion gyroradius. The ion scales are smaller than the grid scale, but not by much. The breakdown most likely occurs with new physics dominating at those scales. We now list some possible sources of dissipation and speculate on how they could be included.

**Table 1.** Length scales in the plasma.

	Length scale	Value ( 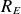 )	
	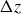	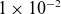	
	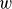	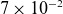	
	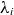	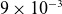	
	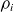	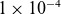	
	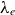	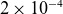	
	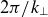	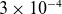	
	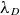	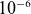	

#### 4.3.2. Possible sources of dissipation

Di Mare & Howes (2024) explain observations of the TRICE-2 rocket in terms of a two-stage cascade. First MHD Alfvén waves cascade in perpendicular wavenumber until 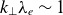 . Second, a parallel cascade begins until 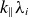 becomes large enough that collisionless damping dominates. Our results show a nonlinearity that becomes significant only when 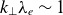 . This feedback of the density causes wave steepening that moves energy to higher 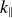 . The mechanism described in this study is consistent with this view of a second stage of the cascade. Perhaps this offers more detail on how this cascade occurs.

The conclusion of viewing our results as the secondary cascade in Di Mare & Howes (2024) is that the missing dissipation in our model comes from collisionless damping sources. Identifying the dominant damping source and including this into the equations of motion are left as future work.

Another idea is to include the curvature drift into the perpendicular dynamics. The curvature drift for a single particle is
(4.13)

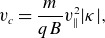

where 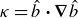 is the curvature of the field line. For a Maxwellian distribution characterised by a single thermal speed and bulk parallel velocity, one can show that the current that results is
(4.14)

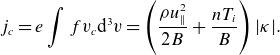




The second term in (4.14) is a thermal finite Larmor radius effect of the ions. It has been discussed in the context of particle acceleration ( 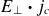 ) in the literature (Beresnyak & Li 2016; Yang *et al*. 2019; Du *et al*. 2022; Ji *et al*. 2022). Beresnyak & Li (2016) and Du *et al*. (2022) approach this from a fluid perspective, so they discount the curvature current from a thermal distribution with no bulk velocity (i.e. they only calculate the first term of (4.14)). Our (4.14) is equivalent to (8) in Ji *et al*. (2022). Using the thermal term with 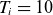 eV, 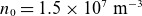 , 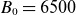 nT and the maximum perpendicular curvature of figure 2, for our system 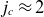 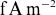 . Using the maximum parallel curvature, 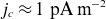 .

**Figure 5.**
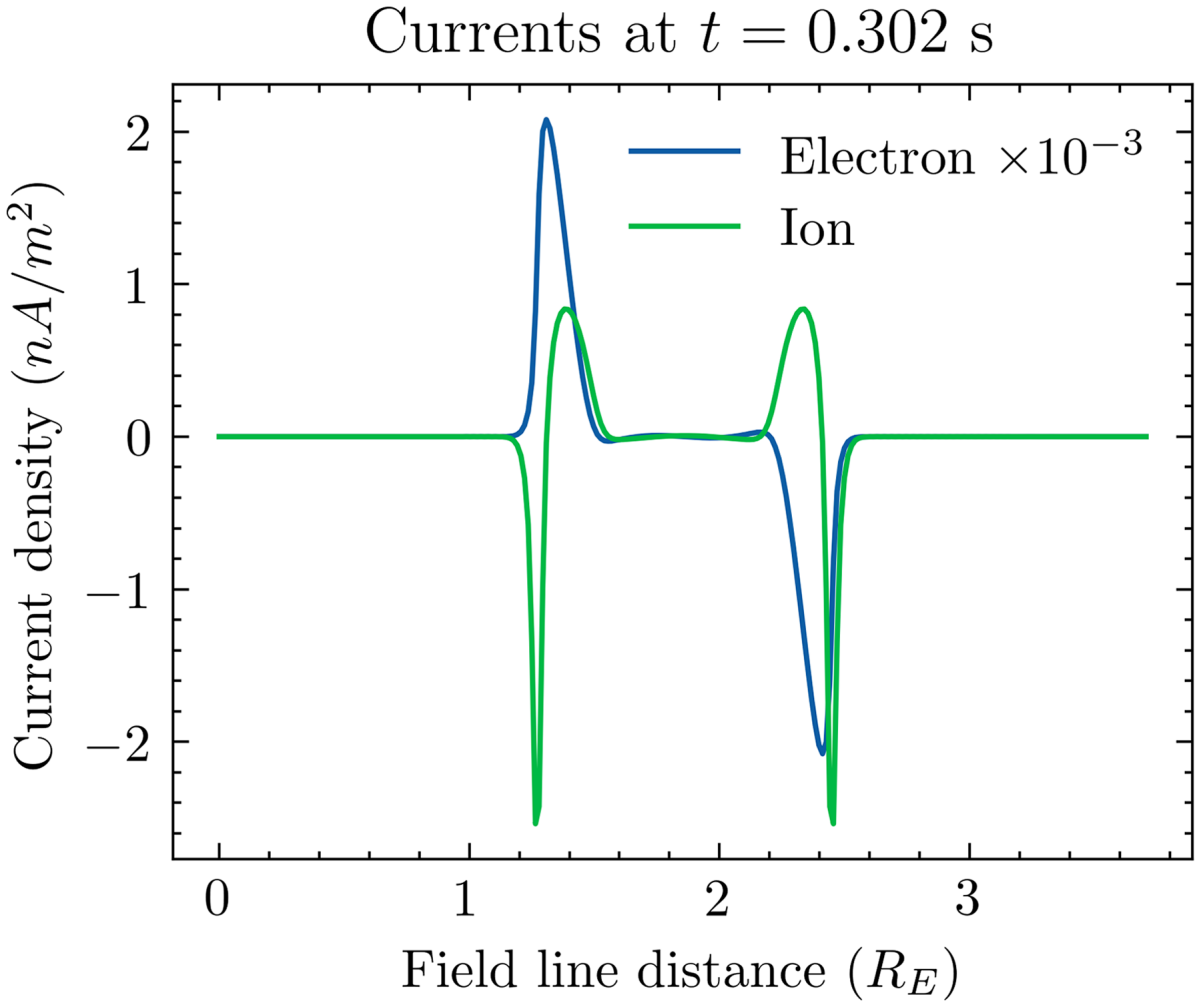
Currents in the system at late time. The electron current has been multiplied by 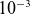 to fit on the same *y* axis as the ion current. The ion current is perpendicular to the field line, while the electron current is parallel to it.

Figure 5 shows the current system at late times. The electron current is the parallel current, also called the field-aligned current. The ion current is the perpendicular current that must exist to close quasi-neutrality. The ion current has been multiplied by 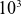 to make it appear on the same axis as the parallel current. The estimated perpendicular curvature current at the discontinuity from figure 2 is still six orders of magnitude lower than the maximum ion current from the polarisation drift. The parallel current is three orders of magnitude less. This suggests that ion cyclotron damping is a stronger effect before curvature drift.

However, if the wave continues to steepen, coupling to the slow-mode compressional MHD wave could become important. This coupling becomes significant when the field line is curved (Southwood & Saunders 1985; Chen & Hasegawa 1991; Hollweg 1999; Kostarev, Mager & Klimushkin 2021). Due to the density perturbations present in inertial Alfvén waves, it seems likely that this physics is pertinent. An interesting area of future work would be to see if this steepening induces coupling to compressional MHD waves. The wave energy would parametrically decay into a smaller Alfvén wave and new slow-mode wave.

## 5. Conclusion and discussion

This study examined steepening of inertial Alfvén waves using a one-dimensional drift-kinetic model where the parallel current driven by the electrons is modelled kinetically and the perpendicular current, driven by the ion polarisation drift, is used to derive an evolution equation for the electrostatic potential in terms of the parallel current. This equation assumes that only a single perpendicular mode is being considered. It was found that such a wave rapidly steepens in the direction of propagation. An analytical time- and length-scale estimate of this steepening was derived (4.5)–(4.8) and shown to agree with the drift-kinetic simulation.

From these analytical estimates, we learn that the steepening increases with 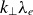 . The smaller the perpendicular wavelength, the faster the wave will steepen. This is because the steepening is due to the Alfvén wave developing a wavelength-dependent density perturbation (3.11). This perturbation is not present when 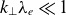 . It has yet to be seen if experiments of Alfvén waves will observe this because its magnitude is quite small. However, it drastically changes the nature of the wave propagation.

Inertial Alfvén waves are thought to be responsible for particle acceleration in the Earth’s ionosphere. The steepening process described in this study predicts that all particle acceleration will happen on the leading edge of the wavefront. When the wave reaches the minimum length scale, which remains an open question, (4.2) would give the apparent width of the electric field. As mentioned in the introduction, several studies have qualitatively observed that the parallel electric field occurs on a ‘shock front’ ahead of the wave. This study gives a theoretical basis as to why this may be.

The Rankine–Hugoniot conditions in § 4.2 demonstrate that at small perpendicular wavelengths, the inertial Alfvén wave begins to act like an electron hydrodynamic shock and less like a magnetic perturbation. The magnetic pressure hardly plays any role in the pressure balance. The electron dynamic pressure that is created from the parallel current in the Alfvén wave is stagnating on the leading edge of the wave when the wave is moving faster than the phase speed. This too is interesting because it demonstrates that when 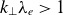 , Alfvén waves begin to have a compressive nature. Wave steepening is a feature of compressive waves.

In addition, this work has a strong connection to recent discoveries in auroral region turbulence. Di Mare & Howes (2024) demonstrate that a secondary turbulent cascade happens between 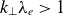 and 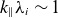 . Our work has shown nonlinear steepening when 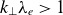 . Table 1 suggests that the next scale happens at 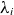 . Perhaps further work will demonstrate that including dissipation physics into the drift-kinetic simulation will produce a similar turbulent cascade.

## Supplementary material

The simulation data and plotting scripts can be found in the supplementary material (DesJardin 2025).
